# Evolution of Movement Disorders in Patients With CLN2-Batten Disease Treated With Enzyme Replacement Therapy

**DOI:** 10.1212/WNL.0000000000209615

**Published:** 2024-07-08

**Authors:** Robert Spaull, Audrey K. Soo, Spyros Batzios, Emma Footitt, Rebecca Whiteley, Jonathan W. Mink, Lucinda Carr, Paul Gissen, Manju A. Kurian

**Affiliations:** From the Molecular Neurosciences (R.S., A.K.S., M.A.K.), Developmental Neurosciences, Zayed Centre for Research into Rare Disease in Children, UCL Great Ormond Street Institute of Child Health, London, United Kingdom; Department of Neurology (R.S., A.K.S., L.C., M.A.K.), Great Ormond Street Hospital, London, United Kingdom; Department of Paediatric Metabolic Diseases (S.B., E.F., R.W., P.G.), Great Ormond Street Hospital for Children, London, United Kingdom; Department of Neurology (J.W.M.), University of Rochester, NY; and Genetics and Genomic Medicine (P.G.), UCL Great Ormond Street Institute of Child Health, London, United Kingdom.

## Abstract

**Objectives:**

Neuronal ceroid lipofuscinosis type 2 (CLN2-disease) is an inherited childhood-onset neurodegenerative condition, with classical early features of speech delay, epilepsy, myoclonus, ataxia, and motor regression. This study aimed to better characterize the spectrum of movement disorders in CLN2-disease in a cohort of children receiving enzyme replacement therapy (ERT).

**Methods:**

A cohort of 18 children attending a single center for treatment with cerliponase alfa ERT was systematically assessed using a standardized structured history and a double-scored, video-recorded examination using the Unified Batten Disease Rating Scale (UBDRS) and Abnormal Involuntary Movement Scale.

**Results:**

Noncanonical movement disorders are common: while ataxia (89%) and myoclonus (83%) were near-universal, spasticity and dystonia were experienced by over half (61% each), with children having a median of 4 distinct movement disorder phenotypes. This progression was stereotyped with initial ataxia/myoclonus, then hyperkinesia/spasticity, and later hypokinesia. ERT slows progression of movement disorders, as measured by the UBDRS physical subscale, with 1.45 points-per-month progression before diagnosis and 0.44 points-per-month while on treatment (*p* = 0.019).

**Discussion:**

Movement disorders are a core feature of CLN2-disease and follow a typical pattern of progression which is slowed by ERT. Identifying and treating movement disorders should become standard, especially given increased patient survival.

## Introduction

Neuronal ceroid lipofuscinoses (NCL) are a group of inherited lysosomal disorders that typically cause progressive neurodegeneration in childhood, with the unifying feature of accumulation of autoflourescent ceroid lipofuscin. CLN2-disease is an NCL caused by biallelic loss-of-function variants in *CLN2/TPP1* leading to reduced synthesis of tripeptidyl peptidase 1 (TPP1).^1^ After a period of either normal development or delayed speech, children present age 2–4 years with epileptic seizures and ataxia, progressing to loss of ambulation, dementia, blindness, and early death.^2-4^ Cerliponase alfa (Brineura, BioMarin) is an enzyme replacement therapy (ERT) delivered by fortnightly intracerebroventricular infusion that prolongs life and slows progression in classical CLN2-disease.^5^

CLN2-disease natural history has been characterized using functional scales, typically either Hamburg (modified to CLN2-Disease Rating Scale) or Weill-Cornell, which focus on seizures, ambulation, language, and visual function.^2,4,6^ Some movement disorders are well-recognized: ataxia is an early hallmark contributing to loss of ambulation; myoclonus is almost universal representing both epileptic and nonepileptic movements.^2^ Other movement disorders such as chorea, tremor, and dystonia are less well characterized.

In this study, we aimed to systematically characterize the range of movement disorders in children with CLN2-disease using cross-sectional standardized clinical assessment and structured data review of a cohort of children receiving ERT at a single center.

## Methods

### Standard Protocol Approvals, Registrations, and Patient Consents

Children with biallelic pathogenic *TPP1* variants and confirmed CLN2-disease attending Great Ormond Street Hospital for regular administration of ERT were recruited. This study was approved by the United Kingdom Research Ethics Service (Research Ethics Committee: London–Bloomsbury: 13/LO/0168). Families provided written consent for inclusion and video recording. The standardized clinical assessments comprised a structured clinical history and review of clinical records and a video-recorded clinical examination using the Unified Batten Disease Rating Scale (UBDRS) and the Abnormal Involuntary Movement Scale (AIMS).^7-10^

### Statistical Analysis

Summary statistics on clinical data were included for 18 children and UBDRS physical subscale (UBDRSp) and AIMS scores for 15 children. Statistical analysis used R (version 4.3.2).^11^ Time-to-event analysis was performed on Kaplan-Meier cumulative incidence curves for each movement phenotype using the survminer package^12^; significance was tested with log-rank tests and Holm correction where multiple comparisons were made. UBDRSp score correlation with age was assessed using the nonparametric Spearman rank; the contribution to this correlation from age at diagnosis, time to ERT start, and time on ERT was assessed using multiple regression in R (see eMethods for full detail).

### Data Availability

All anonymous data have been shared in the eMethods; further patient-level data sharing may be possible by request to the corresponding author subject to legal and ethical considerations.

## Results

### Movement Disorders Are Common

This cohort of 18 children treated with ERT included 10 girls and 8 boys, with a median age of 7 years 1 month, range 5–11.8 years (Table 1). The majority had movement disorders: these included near-universal ataxia (89%) and myoclonus (83%), spasticity (61%) and dystonia (61%) in over half, and later hypokinesia (44%) (Video 1). Only 2 of 18 had not experienced additional movement disorders beyond ataxia and myoclonus with a median of 4 different phenotypes per child (range 0–7). Stereotypies and bruxism were described in 6 of 18 and 3 of 18, respectively (eTable 1).

**Table:** Summary of Clinical Details and Movement Disorders Present or Previously Reported

ID	Sex	Age	Diagnosis (m)	ERT start (m)	ERT duration (m)	Walk	Talk	Epilepsy	Dyst.	Myoc.	Chorea	OLD	Tremor	Ataxia	Hypok.	Spast.	Other	Drugs for MD	UBDRS	AIMS
1	F	8–10	27	47	50	Y	Y	Y	Y	Y	Y	Y	N	Y	N	Y	N	Clonazepam	24	12
2	M	8–10	49	70	52	N	N	Y	Y	Y	N	Y	N	Y	Y	Y	N	Baclofen, botox, clonazepam	68	8
3	M	8–10	95	101	19	Y	Y	N	Y	Y	Y	Y	Y	Y	N	N	N	Nil	40	11
4	M	5–7	51	52	14	N	Y	Y	Y	Y	Y	Y	N	Y	Y	Y	Bruxism, stereotypies	Gabapentin, trihex	53	12
5	F	8–10	55	56	53	N	N	Y	Y	Y	Y	N	Y	N	Y	Y	Torticollis, stereotypies	Baclofen, botox, clobazam, gabapentin	78	3
6	F	5–7	15	21	46	Y	Y	Y	N	N	N	N	N	N	N	N	N	Nil	4	0
7	F	11–13	49	53	89	N	N	Y	Y	N	Y	Y	N	Y	Y	Y	Bruxism, stereotypies	Alimemazine, clonazepam, chloral hydrate, midazolam	59	6
8	M	8–10	50	52	48	Y	N	Y	N	Y	N	N	N	Y	Y	Y	Bruxism, stereotypies	Nil	50	3
9	F	5–7	54	55	17	N	N	Y	Y	N	Y	N	N	Y	N	Y	N	Nil	57	3
10	F	5–7	49	52	21	Y	Y	Y	N	Y	N	N	Y	Y	N	Y	Stereotypies	Nil	34	4
11	F	8–10	45	47	83	N	N	Y	Y	Y	N	Y	Y	Y	Y	Y	Torticollis	Gabapentin	61	9
12	F	5–7	46	47	22	N	N	Y	Y	Y	N	N	N	Y	Y	N	Stereotypies	Clobazam	40	0
13	F	5–7	53	54	11	N	Y	Y	N	Y	Y	Y	Y	Y	N	Y	N	Nil	39	7
14	F	8–10	53	55	57	N	Y	Y	Y	Y	Y	Y	Y	Y	N	N	N	Baclofen, diazepam, gabapentin	43	20
15	M	11–13	47	52	85	N	N	Y	Y	Y	Y	N	N	Y	Y	Y	N	Clonazepam	17	0
16	M	5–7	57	58	7	Y	Y	Y	N	Y	N	N	Y	Y	N	N	N	Nil	—	—
17	M	5–7	40	51	9	N	Y	Y	N	Y	N	N	N	Y	N	N	N	Nil	—	—
18	M	5–7	62	63	1	Y	Y	Y	N	Y	N	N	Y	Y	N	N	N	Nil	—	—

Abbreviations: AIMS = Abnormal Involuntary Movement Scale score (possible range from 0 (unaffected) to 24); ERT = enzyme replacement therapy; Dyst. = dystonia; Hypok. = hypokinesia; Myoc. = myoclonus; MD = movement disorder; OLD = orolingual dyskinesia; Spast. = spasticity; trihex. = trihexyphenidyl; UBDRS = Unified Batten Disease Rating Scale physical subscale score (possible score range from 0 (unaffected) to 112).

10.1212/209615_Video_1 Video 1 Movement disorder phenotypes in the CLN2 patient cohort. Movement disorder phenotypes in the CLN2 patient cohort, extracted from the video examinations: ataxia evidenced by a broad-based gait and tendency to fall, with added left foot dystonic posturing; brief generalized myoclonus at rest; marked bilateral upper limb action tremor on reaching out; generalized chorea while seated in a car seat; hyperreflexia with spreading and sustained ankle clonus; bilateral dystonic great toe posturing; and generalized hypokinesia.Download Supplementary Video 1 via http://dx.doi.org/10.1212/209615_Video_1

Cross-sectional examination included indicators of severity according to the UBDRS or AIMS descriptors, ranging from 0-none to 4-severe. Dystonia was present in 9 of 15 (2 minimal, 2 mild, 5 moderate), spasticity in 7 of 15 (worst limb score: 4 minimal, 1 mild, 2 moderate), chorea in 6 of 15 (1 minimal, 3 mild, 2 severe), and hypokinesia in 7 of 15 (1 minimal, 2 mild, 4 moderate). (eTable 2).

### Movement Disorders Progress in a Typical Pattern

Children with CLN2-disease follow a typical pattern of progression of their movement disorder. Figure 1A shows a time-to-event analysis with cumulative probability of developing the most common phenotypes: median age at ataxia onset is 4 years, myoclonus 5 years, spasticity 7.5 years, dystonia 8 years, and hypokinesia 10 years (log-rank test of differences *p* = 0.0014). Figure 1B shows phenotype progression in relation to ages at diagnosis and ERT commencement for each individual.

**Figure:**
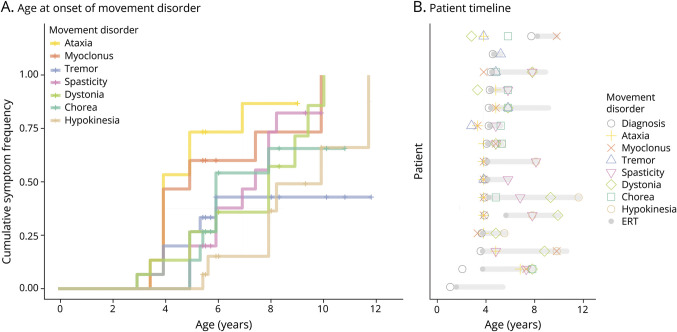
Movement Disorder Presentation and Phenotypes (A) Kaplan-Meier cumulative probability curves of developing the commonest movement disorders present in this group against age. This shows the high prevalence of each phenomenon, progressing over time: ataxia and myoclonus (median onset age 4 and 5 years, respectively), spasticity and dystonia (median onset age 7.5 and 8 years, respectively), and hypokinesia (median onset age 10 years). Tick marks across each line indicate statistical censoring of data where individuals had not yet developed the phenotype at their last assessment and so were not contributing to the probability analysis for higher ages. (B) Individual patient timelines, ordered by decreasing age at diagnosis of CLN2-disease, with the gray bar indicating continued receipt of ERT.

### ERT Slows Worsening of UBDRS Score and Movement Disorder Progression

UBDRSp scores, which comprises function and movement disorder severity, increase with age at assessment (*r* = 0.63, *p* = 0.012). Multiple regression analysis showed a greater contribution from age at diagnosis (1.45 UBDRSp-points-per-month before diagnosis, *p* = 0.003) compared with while receiving ERT (0.44 UBDRSp-points-per-month on ERT, *p* = 0.019) (eMethods). This indicates that progression slows but does not stop after starting ERT. There was no apparent correlation with other variables such as sex or common genotype.

### Treatment for Movement Disorders Is Variable

Only half of the cohort had received medications or treatments for movement disorders, despite 9 of 15 having at least one moderate/severe movement disorder noted on examination. On average, 2 medications had been trialed per patient (range 0–4); gabapentin and clonazepam were most commonly used (5 and 4 patients, respectively) (Table 1). Parents reported that gabapentin was generally effective for dystonia [mean dose 43 mg/kg/d, range 26–60 mg/kg/d], but less consistent benefit was reported for other medications. Medication-related worsening of symptoms was not reported, and side effects were rarely reported (drowsiness with gabapentin, n = 2; reduction in central tone with baclofen, n = 1).

## Discussion

This cross-sectional assessment of movement disorders in a cohort of children with CLN2-disease receiving ERT shows that movement disorders are near-universal, follow a typical pattern of development, and that ERT appears to slow progression. This systematic analysis corroborates and expands on previously described frequent pyramidal signs and infrequent chorea, tremor, and dystonia in ERT-naive children.^2^ It is likely that these movement disorders are part of the natural history of CLN2-disease, rather than a consequence of treatment, and predominantly reflect disease progression *before* starting ERT. Even when ERT is started, it can take several months to see improvement in CLN2 scores and biomarkers.^13^ It is also possible that ERT is prolonging lifespan, which may alter the natural history of disease, resulting in a new “ERT-treated disease phenotype” including a spectrum of movement disorders. Although intracerebroventricular delivery of recombinant TPP1 in dogs led to widespread CNS penetration including key areas for motor control,^14^ future data on the biodistribution of ERT in patients will be informative for understanding which regions of the brain are effectively targeted.

The progression of movement symptoms from ataxia and myoclonus (median age 4–5 years) through to both pyramidal (spasticity) and extra-pyramidal (dystonia) features (age 8 years) and then to hypokinesia (age 10 years), indicates progressive functional impairment. Early myoclonus may be cortical or subcortical in origin,^3,4^ but often accompanies the onset of generalized convulsive seizures. Loss of ambulation may initially be related to ataxia but also negative myoclonus and later spasticity and dystonia. This stereotyped progression bears some similarity to other NCLs and neurometabolic disorders such as juvenile Tay-Sachs disease.^15^ Whether the observed hypokinesia is parkinsonian remains to be determined, as other features such as bradykinesia and rigidity are not seen.

Qualitative assessment (Figure 1B) supported by multiple regression analysis indicates slower progression on ERT, suggesting that progression of movement disorders slows with treatment, in keeping with the slower overall disease progression on ERT.

Notably, 3 patients (03, 09, 13) presented atypically with a movement disorder other than myoclonus or ataxia before epilepsy: 2 with dystonia and another with tremor. Two (03, 14) have a predominantly hyperkinetic phenotype, with marked choreiform and ballistic movements that are treatment-refractory. With increasing availability of genomic testing, enzyme assay, and ERT, CLN2-disease should be considered early in the diagnostic pathway for children with movement disorders.

This study is limited in assessment of natural history, as all participants started ERT at varying disease stages as it became available. Given that ERT slows disease progression, it is likely that this study underestimates the burden of movement disorders in untreated disease. In addition, our ERT access program excluded children with very severe disease, which might have led to study bias by negative selection.

In conclusion, this study provides an in-depth assessment of movement disorders associated with CLN2-disease in a cohort of children receiving ERT. While ataxia and myoclonus have long been hallmarks of early CLN2-disease, other movement disorder phenotypes have received less focus. In this era of disease-modifying treatment and increased survival, a more holistic approach to management and treatment can be informed by this assessment of phenotype progression that will better maintain quality of life, inclusion, and functional attainment.

## Appendix Authors

**Table:**

Name	Location	Contribution
Robert Spaull, MA, MBBS, MRCPCH	Molecular Neurosciences, Developmental Neurosciences, Zayed Centre for Research into Rare Disease in Children, UCL Great Ormond Street Institute of Child Health; Department of Neurology, Great Ormond Street Hospital, London, United Kingdom	Drafting/revision of the manuscript for content, including medical writing for content; major role in the acquisition of data; study concept or design; analysis or interpretation of data
Audrey K. Soo, BSc, MBBS, PhD	Molecular Neurosciences, Developmental Neurosciences, Zayed Centre for Research into Rare Disease in Children, UCL Great Ormond Street Institute of Child Health; Department of Neurology, Great Ormond Street Hospital, London, United Kingdom	Drafting/revision of the manuscript for content, including medical writing for content; major role in the acquisition of data
Spyros Batzios	Department of Paediatric Metabolic Diseases, Great Ormond Street Hospital for Children, London, United Kingdom	Drafting/revision of the manuscript for content, including medical writing for content
Emma Footitt, BSc, MBBS, PhD	Department of Paediatric Metabolic Diseases, Great Ormond Street Hospital for Children, London, United Kingdom	Drafting/revision of the manuscript for content, including medical writing for content
Rebecca Whiteley, BA	Department of Paediatric Metabolic Diseases, Great Ormond Street Hospital for Children, London, United Kingdom	Major role in the acquisition of data
Jonathan W. Mink, MD, PhD, FAAN	Department of Neurology, University of Rochester, NY	Drafting/revision of the manuscript for content, including medical writing for content; study concept or design
Lucinda Carr, MBChB, MD, FRCPCH	Department of Neurology, Great Ormond Street Hospital, London, United Kingdom	Drafting/revision of the manuscript for content, including medical writing for content; study concept or design; analysis or interpretation of data
Paul Gissen, MD, PhD	Department of Paediatric Metabolic Diseases, Great Ormond Street Hospital for Children; Genetics and Genomic Medicine, UCL Great Ormond Street Institute of Child Health, London, United Kingdom	Drafting/revision of the manuscript for content, including medical writing for content; study concept or design
Manju A. Kurian, MA, MBBChir, MRCPCH, PhD	Molecular Neurosciences, Developmental Neurosciences, Zayed Centre for Research into Rare Disease in Children, UCL Great Ormond Street Institute of Child Health; Department of Neurology, Great Ormond Street Hospital, London, United Kingdom	Drafting/revision of the manuscript for content, including medical writing for content; study concept or design; analysis or interpretation of data
